# Malformation of the Female Genitals

**Published:** 1898-12

**Authors:** Calvin Carter

**Affiliations:** Brooksville, Ind.


### MALFORMATION OF THE FEMALE GENITALS.

BY CALVIN CARTER, M.S., M.D., BROOKSVILLE, IND.

Subject: aged sixteen years; nationality, American; temperament, sanguine; complexion, blonde; well developed and decidedly muscular; morals, lewd, can hardly be classed as a prostitute; sexual desire, above the average. First seen September 10, 1898. When asked how long she had been guilty of sexual relations, replied, "Since fourteen years of age." When the parts were exposed, I was amazed to see protruding from the pudendum, and hanging down after the fashion of the flaccid penis, an object of malformation. Placing the patient in the lithotomy position, we exposed the parts, and were about finding out what abnormality we had to deal with. Investigation soon dispelled the idea of hermaphroditism. The pubes are perfectly normal, the labia majora are normal. The clitoris stands up prominently, and has a distinct glans. Unless the malformation under consideration is to be classed as the libia minora, these folds are absent in this subject. The lines leading up from the labia minora to the under side of the clitoris, and the lines or folds leading from the glans to the labia minora, are absent. The malformation under consideration, in the flaccid or normal state, extends to within about a half-inch of the posterior border of the orifice to the vagina. It begins to develop at this point, and extends to and is continuous with the integument beneath the clitoris, and from side to side of the orifice of the vagina, covering it almost completely as with an apron. Gentle traction will bring the border down to the opening of the rectum. Placing the finger beneath and pressing upward, it balloons until it will measure a little more than five inches from lowest point of attachment to opposite. The lower margin is regular, with the exception of one notch in the center, extending upward toward the clitoris of about one inch on the stretch, and not particularly noticeable when in the flaccid condition. That part of the malformation which is exposed to the air is covered with integument resembling the skin. The under surface has the general appearance of the mucous membrane of the vagina. The urethra is about two-thirds the length usually found in women. The meatus arinarius is placed well under the public bones, at least three-quarters inch farther back than usually found. A gentle pressure of the malformation between the finger and thumb leaves the sensation that is present when pressing the scrotum of a male in like manner. The subject says the malformation is the principal source of pleasure in the act of copulation. She strenuously denies that the malformation can be a result of manipulation, and claims that she has been aware of its existence for a good many years.—*Ohio Medical Journal.*

